# Revision surgery after rod breakage in a patient with occipitocervical fusion: A case report

**DOI:** 10.1097/MD.0000000000010441

**Published:** 2018-04-13

**Authors:** Chao Tang, Guang Zhou Li, Min Kang, Ye Hui Liao, Qiang Tang, De Jun Zhong

**Affiliations:** a Department of Spine Surgery; b Department of Gastroenterology, Affiliated Hospital of Southwest Medical University, China.

**Keywords:** occipitocervical angle, occipitocervical fusion, revision surgery, rod breakage

## Abstract

**Rationale:**

Rod breakage after occipitocervical fusion (OCF) has never been described in a patient who has undergone surgery for basilar invagination (BI) and atlantoaxial dislocation (AAD). Here, we present an unusual but significant case of revision surgery to correct this complication.

**Patient concerns:**

A 32-year-old female presented with neck pain, unstable leg motion in walking, and also BI with AAD. Her first surgery was planned to correct these conditions and for fusion at the occipital junction (C3-4) using a screw-rod system. At the 31-month follow-up after her first operation, the patient complained of severe neck pain and limitation of motion, suggesting rod breakage.

**Diagnoses:**

Rod breakage after occipitocervical fusion for BI and AAD.

**Interventions:**

The patient underwent reoperation for replacement of the broken rods, adjustment of the occipitocervical angle, maintenance of the bone graft bed, and fusion.

**Outcomes:**

At follow-up, the hardware was found to be in good condition, with no significant loss of cervical lordosis. At the 37-month follow-up after her second operation, the patient was doing better and continuing to recover.

**Lessons:**

We concluded that nonideal choice of occipitocervical angle may play an important role in rod breakage; however, an inadequate bone graft and poor postoperative fusion may also contribute to implant failure.

## 1 Introduction

Occipitocervical fusion (OCF) is an effective surgical treatment for trauma, inflammation, tumor, and congenital diseases of the spine, and also iatrogenic factors leading to occipitocervical instability.^[1–3]^ In the treatment of occipitocervical diseases such as basilar invagination (BI) and atlantoaxial dislocation (AAD), OCF has produced satisfactory clinical results.^[4–6]^ However, rod breakage is a common complication after spinal fusion surgery. In their study, Smith et al^[7]^ found a global incidence of symptomatic rod breakage of 6.8% in adult patients who underwent corrective surgery for spinal deformities. Okamoto et al^[8]^ reported an implant failure rate of 4.2% (6/142) after posterior cervical spine fusion, including occipital plate fracture, disassembly of the pedicle screw and rod, and breakage of Magerl and cervical pedicle screws. However, there have been no reports of rod breakage after the treatment of these conditions or in BI with AAD after occipitocervical fusion. In this report, we present a case of revision surgery in a female patient who experienced rod breakage after occipitocervical fusion.

## 2 Case report

**Figure 1:**
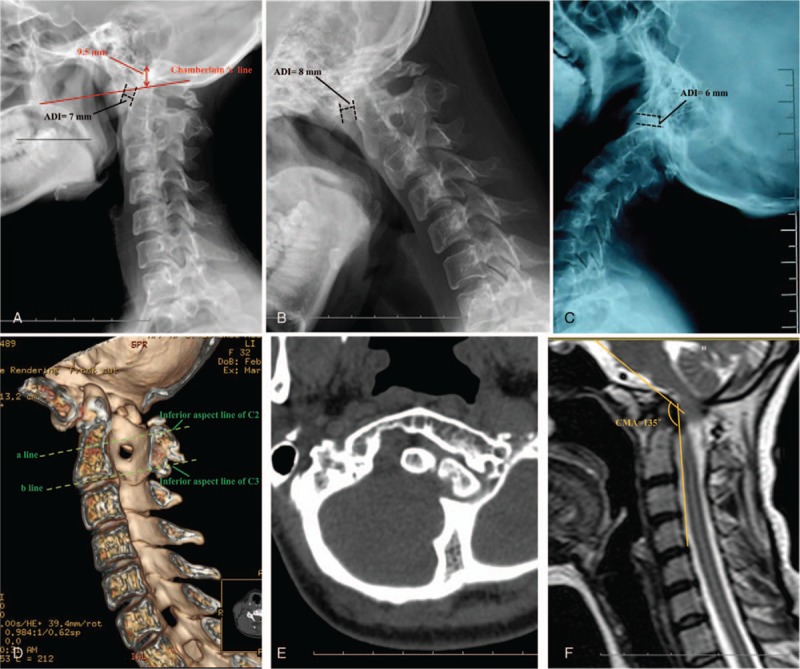
**(**A–C) X-ray taken in the lateral neutral position before surgery, showing the high position of the odontoid and atlantoaxial dislocation with an occipital-C2 angle (OC2A) of 5.1 degrees and a posterior occipitocervical angle (POCA) of 130.6 degrees. (D, E) Cervical 3-dimensional computed tomography reconstruction revealing an atlanto-occipital malformation and C2-3 fusion. Line (a) indicates the inferior aspect line of C2; line (b) indicates the inferior aspect of C3; these 2 lines are parallel. ADI = atlas-dens interval, CMA = cervicomedullary angle (angle subtended by the lines drawn parallel to the ventral surfaces of the medulla and upper cervical cord), OC2A = occipital-C2 angle (angle between McGregor line and the line tangential to the inferior aspect of the axis), POCA = posterior occipitocervical angle (angle formed by the intersection of a line drawn tangential to the flat posterior aspect of the occiput and the line determined by the posterior aspect of the third and fourth cervical facets).

We present the case of a 32-year-old woman who was diagnosed with BI and AAD. She remained untreated until the spinal cord compression became sharply worse, causing unstable walking and severe neck pain. At her first outpatient visit, plain cervical radiographs showed the following in the sagittal plane: a 9.5-mm distance from the odontoid tip to Chamberlain line and an atlas-dens interval (ADI) of 7 mm (Fig. 1), suggesting the need for surgical correction.

After the patient was hospitalized, further examination showed obvious neck tenderness, increased muscle tension in the lower limbs, and hyper-reactivity of knee and ankle flexion. The pathologic sign was positive.

**Figure 2:**
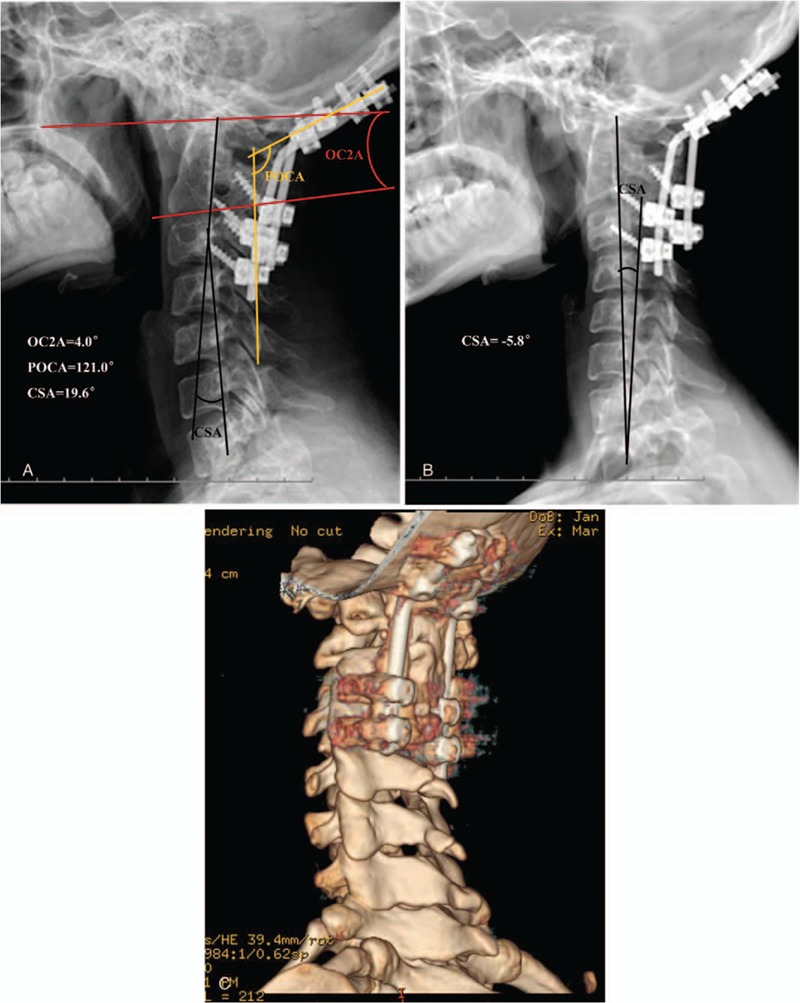
(A) X-ray taken in the lateral neutral position soon after the first surgery and ambulation. (B, C) Imaging data from 13-month follow-up after the first surgery. X-ray indicates subaxial kyphosis with a cervical spine angle (CSA) of −5.8 degrees. Computed tomography reconstruction shows incomplete fusion at the occipitocervical junction. CSA =  cervical spine angle (angle between the posterior aspects of vertebral bodies C2 and C7).

On imaging examination, x-ray showed the odontoid in a high position, and also atlantoaxial dislocation. Flexion and extension plane ADI were 8 and 6 mm, respectively. The occipital-C2 angle (OC2A) and posterior occipitocervical angle (POCA) were 5.1and 130.6 degrees, respectively. Cervical 3-dimensional (3D) computed tomography (CT) reconstruction indicated an atlanto-occipital malformation and C2-3 fusion, leading to difficulty in locating the tangent of the inferior aspect of C2. We replaced the inferior aspect line of C2 by C3 order to measure OC2A, because these lines are parallel in sagittal CT reconstruction (Fig. 1). Magnetic resonance imaging revealed a cervicomedullary angle of 135 degrees. On April 11, 2012, posterior correction, fixation, and fusion were performed as follows: under general anesthesia, satisfactory restoration of the upper cervical dislocation by strong (8 kg) cranial traction; full exposure of the C1-4 bilateral vertebral lamina and bilateral occipital parts of about 3 cm. Four occipital screws were implanted in the bilateral occipital plate and lateral mass screws were implanted in each vertebra (C3, C4). Suitable rods were molded into a normal spinal curve using a plate bender. A cantilever technique was used during rod installation. Posterolateral and occipital plate bone graft fusion was performed with autogenous bone. The bleeding volume was approximately 200 mL and somatosensory evoked potentials were elicited during the operation. The cervical neutral sagittal plane postoperatively and soon after surgery with ambulation showed an occipitocervical angle with an OC2A and POCA of 4.0 and 121 degrees, respectively. The distance from the odontoid tip to Chamberlain line was reset to 4 mm with an ADI of 4.5 mm. Bone graft fusion in the occipital plate and facet joints was good, but it was poor in the region of the occipitocervical junction. At the 13-month postoperative follow-up, cervical kyphosis was found in the sagittal plane (Fig. 2). Relative clinical efficacy and occipitocervical angle parameters are shown in Table 1.

**Table 1:**
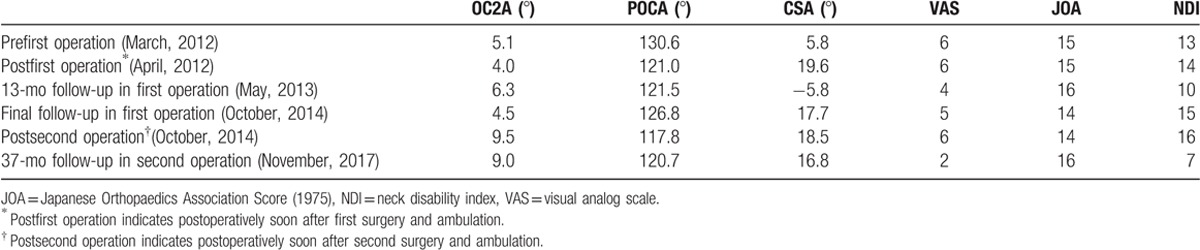
Parameters and clinical efficacy of patient.

**Figure 3:**
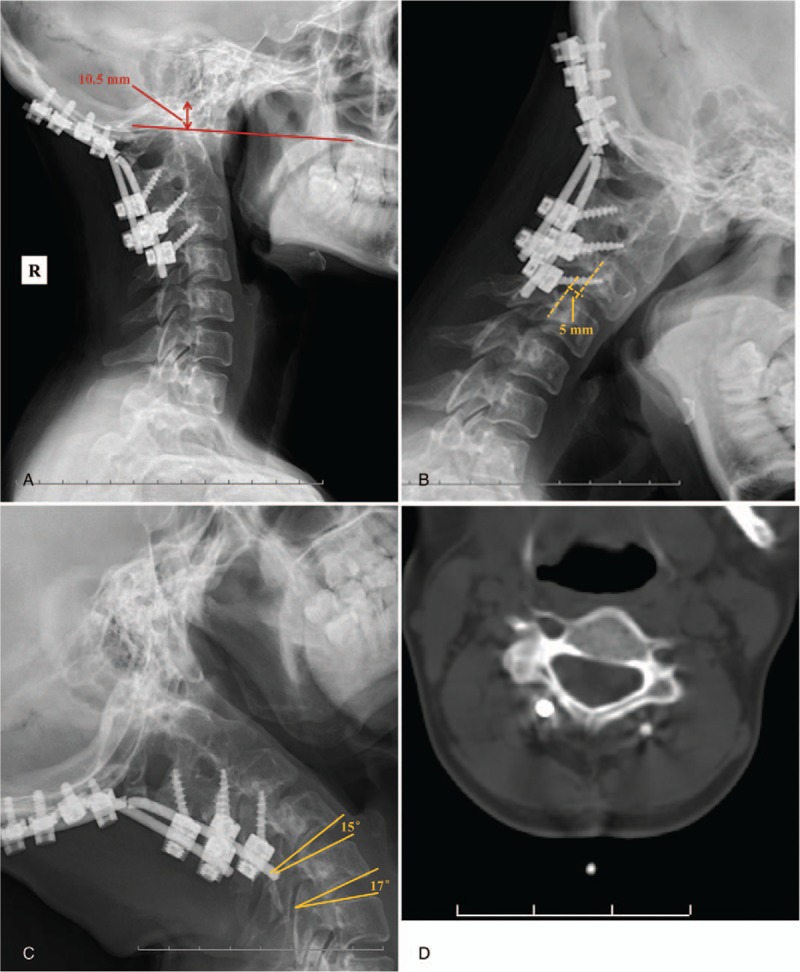
(A) Plain cervical radiographs taken at 31-month follow-up after the first operation showing breakage of the right rod in the region of the occipitocervical junction. (B, C) Lateral x-rays in flexion-extension positions show subaxial cervical instability. (D) Computed tomography scan indicates inadequate fusion of bone graft.

On October 15, 2014, although she had not experienced any trauma, the patient had increasingly severe neck pain (beginning in the previous month). She then returned as an outpatient, and cervical plain radiographs showed right rod breakage in the region of the craniocervical junction; at that time the distance from the odontoid tip to Chamberlain line was 10.5 mm. Cervical 3D CT reconstruction showed poor fusion of the bone graft (Fig. 3). We then suggested that the patient undergo revision surgery.

**Figure 4:**
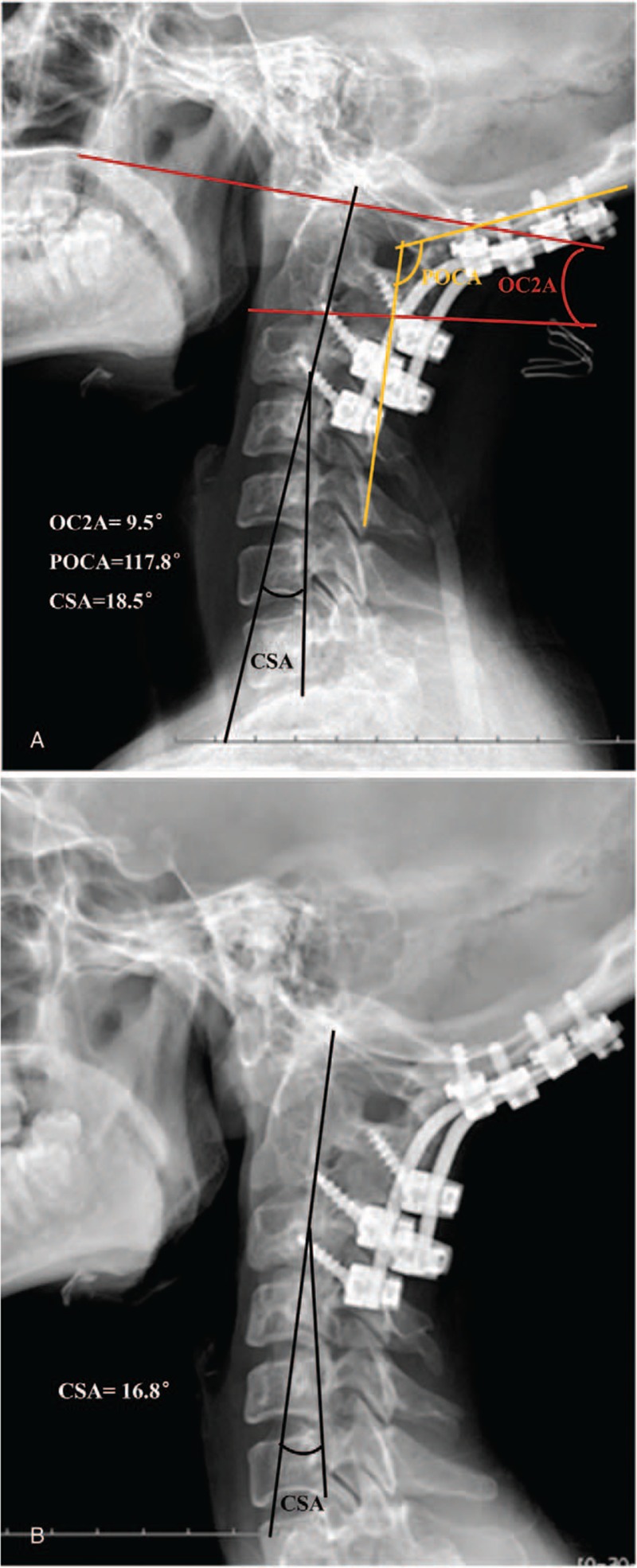
(A) Lateral x-ray after revision surgery showing correct position with an occipital-C2 angle (OC2A) of 9.5 degrees and posterior occipitocervical angle of 117.8 degrees. (B) Lateral x-ray at follow-up 37 months after revision surgery.

On October 18, 2014, a posterior revision was performed. First, the broken rods were removed. Second, suitable rods were installed and the balance of the craniocervical junction was restored. Third, cortical bone was removed and prepared for grafting, whereas unicortical iliac bone graft struts and—after meticulous decortication and resection of the articular cartilage—morcellized bone chips were placed on laminae, facet joints, and occipital bone. Spinal cord monitoring was performed during surgery. The bleeding volume was approximately 300 mL. During surgery, we discovered a rod breakage in the region of the occipitocervical junction, atlanto-occipital joint instability, and failure of the initial fusion. Plain radiographs showed the following in the sagittal plane: satisfactory reduction in the atlanto-occipital region, with OC2A and POCA angles of 9.5 and 117.8 degrees, respectively (Fig. 4). At the 37-month follow-up after the second operation, the hardware was in good condition and the cervical lordosis was significantly intact (Fig. 4). A satisfactory clinical outcome was observed. The relative parameters are listed in Table 1.

## 3 Discussion

In 1910, Pilcher^[9]^ first reported treatment of an atlantoaxial dislocation by occipitocervical fixation surgery. Subsequently, in 1927, Forrester described atlantoaxial and occipitocervical fusion for an unstable odontoid fracture with a fibular onlay graft, which led to satisfactory clinical results. With increasing research on occipitocervical fusion, there have been many reports of complications, such as postoperative neck stiffness, axial pain, dysfunctional neck movement and dizziness, severe dysphagia, and dyspnea.^[10–15]^ In long-term follow-up, the loss of lower cervical lordosis and acceleration of degeneration, even the failure of implants, have been suggested.^[1,8,12]^ At present, many reports indicate that improper OC2A angles in occipitocervical fixation and fusion can affect clinical efficacy and accelerate degeneration of the lower cervical spine.^[16–20]^ Therefore, it is imperative that such a nonideal occipitocervical angle should be avoided, as it can lead to breakage of the implants after occipitocervical fusion.

Although posterior occipitocervical fusion using screw-rod devices has become a popular technique for the treatment of spinal instability or deformity, no studies have discussed rod breakage following the use of these devices. Many researchers have reported on the factors leading to rod breakage after pedicle subtraction osteotomy (PSO). According to the authors’ experience, both insufficient correction of the sagittal plane and great instability at the PSO level—due to the combination of posterior bony discontinuity and anterior mechanical imbalance—may create a massive concentration of stress, causing fatigue fracture of the implant.^[21–23]^ Okamoto et al^[8]^ reported that bone fusion is by far the most important factor in preventing implant failure. Berjano et al suggested that insufficient correction leads to sagittal imbalance and increased mechanical stress on the posterior implant. Tensile forces through the posterior graft cause bone resorption and reduce the chance of obtaining solid fusion.^[24]^

However, the case described here represents an exception—rod breakage after occipitocervical fusion. The patient had symptoms of neck pain and unstable walking before first operation, with visual analog scale (VAS), Japanese Orthopaedics Association Score (1975, JOA), and neck disability index (NDI) of 6, 15, and 13, respectively. The patient was clearly diagnosed as having BI and AAD; occipitocervical fixation and fusion were then performed, establishing an OC2A of 4.0 degrees and a POCA of 121.0 degrees. The increasingly severe neck pain (not due to trauma), however, seriously affected the patient's quality of life after 31 months postsurgery. In particular, the right-sided connecting rods broke at the level of the occipitocervical junction. The distance from the odontoid tip to Chamberlain line increased to 10.5 mm (it was 4.0 mm after the first surgery), and subaxial cervical instability in flexion-extension increased. These findings demonstrate a serious loss of balance and vertebral instability in the region of the occipitocervical junction and cervical sagittal plane. We were puzzled as to the cause of the rod breakage. Was it just a simple matter of poor fusion of the bone graft? What is the ideal occipitcervical angle? Is it OC2A 4 degrees and POCA 121.degrees? Wang et al^[25]^ have indicated that the position of fixation of the OC2A, to maintain the balance of the craniocervical region and decrease the long-term effects on the middle and lower cervical vertebrae, should be in the range of 9 to 22 degrees during occipitocervical fusion. Riel et al^[26]^ suggested that 80% of the POCA value should be between 101 and 119 degrees for normal individuals.

Insufficient correction may have been the most important factors causing the rod to break. It has also been reported that a nonideal correction can lead to sagittal imbalance and increased mechanical stress on the posterior implant after PSO. On the contrary, stress concentration through the posterior graft can cause bone resorption and reduce the chance of obtaining solid fusion. Rod breakage, as a consequence, occurred at the point of stress concentration and the area of poor bone graft fusion. In this case, the patient's imbalance of in the occipitocervical region was corrected with an OC2A of 4 degrees and a POCA of 121 degrees in the first surgery. Poor bone graft fusion in the area of the occipitocervical junction and instability in the lower cervical region became evident at the postsurgical follow-up. Furthermore, subaxial kyphosis and an unsatisfactory clinical outcome were observed. Yoshida et al^[13]^ found that there was a negative correlation between DO-C2 (change in the OC2A) and Dsubaxial lordosis angles (change in subaxial lordosis), suggesting that care must be taken to avoid excessive OC2A correction, since this might induce a mid- to lower cervical compensatory decrease in lordosis. Berjano et al^[24]^concluded that the various methods of compensating for imbalance were not sufficient to free patients from pain because muscular fatigue causes pain over the long term, and the progression of sagittal imbalance can lead to the mobilization or breakage of the implant.

Our patient, whose hardware remained in good condition, obtained a satisfactory clinical outcome, and the cervical lordosis had not been lost at 37-month follow-up postrevision. During the second operation, full bone graft fusion was achieved by unicortical iliac bone graft struts and morcellized bone chips. In addition, the OC2A and POCA were corrected to 9.5 and 117.8 degrees, respectively. Whether a nonideal OC2A and/or POCA will increase the incidence of adverse events postoperatively remains uncertain. There is a lack of information in the literature on this subject. In our opinion, a nonideal angle of the OC2A and/or POCA, establishing occipitalcervical imbalance and a concentration of stress, was the most likely reason for the rod breakage in our patient. On one hand, good bone graft fusion at occipital scale, laminae, and facet joints, expect the region of occipitocervical junction. On the contrary, the VAS (2), JOA (16), and NDI (7) were better than at follow-up after the first operation. In addition, there was no cervical kyphosis or instability at follow-up after the revision surgery. We are confident that the risk can be reduced if this last consideration is kept in mind. We are, in fact, gratified to note that in recent years more and more researchers are paying close attention to the influence of the occipitocervical angle. Unfortunately, there is no clear gold standard for establishment of the ideal occipitocervical angle for occipitocervical fusion. Further study will have to focus on the angles that may cause failure, including both the OC2A and POCA.

## 4 Conclusions

In conclusion, as is commonly recognized, bone graft fusion is an important factor in preventing implant failure. Furthermore, we hypothesized that a nonideal occipitalcervical angle, a key factor in implant failure, could lead to occipitocervical imbalance and thus concentrate stress at in the region of the craniofacial junction. We suggest that it is necessary and important to give close attention to the positions of the fixed occipital bone and axis during procedures of occipitoaxial fusion.

## Acknowledgments

The authors wish to thank the patient for agreeing to the publication of this case report and for permission to use of the images taken during her outpatient and inpatient care.

## Author contributions

**Conceptualization:** De Jun Zhong.

**Data curation:** Chao Tang, Ye Hui Liao, Qiang Tang.

**Formal analysis:** Chao Tang, Min Kang, Ye Hui Liao, De Jun Zhong.

**Investigation:** Chao Tang, Guang Zhou Li.

**Methodology:** Chao Tang, Guang Zhou Li.

**Project administration:** De Jun Zhong.

**Software:** Min Kang.

**Supervision:** Guang Zhou Li, De Jun Zhong.

**Writing – original draft:** Chao Tang.

**Writing – review & editing:** Chao Tang, Guang Zhou Li.

## References

[1] Matsunaga S Ijiri K Koga H Results of a longer than 10-year follow-up of patients with rheumatoid arthritis treated by occipitocervical fusion. Spine 2000;25:1749–53. 10888940 10.1097/00007632-200007150-00002

[2] Matsunaga S Sakou T Onishi T Prognosis of patients with upper cervical lesions caused by rheumatoidarthritis: comparison of occipitocervical fusion between c1 laminectomy and nonsurgical management. Spine 2003;28:1581–7. 12865848

[3] Lee SC Chen JF Lee ST Clinical experience with rigid occipitocervical fusion in the management of traumatic upper cervical spinal instability. J Clin Neurosci 2006;13:193–8. 16459085 10.1016/j.jocn.2005.03.031

[4] Ding X Abumi K Ito M A retrospective study of congenital osseous anomalies at the craniocervical junction treated by occipitocervical plate-rod systems. Eur Spine J 2012;21:1580–9. 22547213 10.1007/s00586-012-2324-x PMC3535228

[5] Bhatia R Desouza RM Bull J Rigid occipitocervical fixation: indications, outeonles, and complications in the modern era. J Neurosurg Spine 2013;18:333–9. 23432328 10.3171/2013.1.SPINE12645

[6] Garrido BJ Sasso RC Occipitocervieal fusion. Orthop Clin North Am 2012;43:1–9. 22082624 10.1016/j.ocl.2011.08.009

[7] Smith JS Shaffrey CI Ames CP Assessment of symptomatic rod fracture after posterior instrumented fusion for adult spinal deformity. Neurosurgery 2012;71:862–8. 22989960 10.1227/NEU.0b013e3182672aab

[8] Okamoto T Neo M Fujibayashi S Mechanical implant failure in posterior cervical spine fusion. Eur Spine J 2012;21:328–34. 22002474 10.1007/s00586-011-2043-8 PMC3265582

[9] Pilcher LS Atlo-axoid fracture: dislocation. Ann Surg 1910;51:208–11. 17862488 10.1097/00000658-191002000-00005 PMC1405924

[10] Matsunaga S Onishi T Sakou T Significance of occipitoaxial angle in subaxial lesion after occipitocervical fusion. Spine 2001;26:161–5. 11154536 10.1097/00007632-200101150-00010

[11] Logroscino CA Genitiempo M Casula S Relevance of the cranioaxial angle in the occipitocervical stabilization using an original construct: a retrospective study on 50 patients. Eur Spine J 2009;18:7–12. 19399534 10.1007/s00586-009-0985-x PMC2899607

[12] Taigo I Takeo F Koshiro K Postoperative increase in occiput-C2 angle negatively impacts subaxial lordosis after occipito-upper cervical posterior fusion surgery. Asian Spine J 2016;10:744–7. 27559456 10.4184/asj.2016.10.4.744 PMC4995259

[13] Yoshida M Neo M Fujibayashi S Upper-airway obstruction after short posterior occipitocervical fusion in a flexed position. Spine 2007;32:E267–270. 17426623 10.1097/01.brs.0000259977.69726.6f

[14] Masanori I Masashi N Mitsuru T The O-C2 angle established at occipito- cervical fusion dictates the patient's destiny in terms of postoperative dyspnea and/or dysphagia. Eur Spine J 2014;23:328–36. 23982903 10.1007/s00586-013-2963-6 PMC3906459

[15] Veena S Rebecca M Pirjo M Airway adverse events following posterior occipito-cervical spinal fusion. J Clin Neurosci 2017;39:124–9. 28110925 10.1016/j.jocn.2016.12.036

[16] Matsunaga S Onishi T Significance of occipitoaxial angle in subaxial lesion after occipitocervical fusion. Spine (Phila Pa 1976) 2001;26:161–5. 11154536 10.1097/00007632-200101150-00010

[17] Passias PG Wang S Kozanek M Relationship between the alignment of the occipitoaxial and subaxial cervical spine in patients with congenital atlantoxial dislocations. J Spinal Disord Tech 2013;26:15–21. 21959834 10.1097/BSD.0b013e31823097f9

[18] Grob D Frauenfelder H Mannion AF The association between cervical spine curvature and neck pain. Eur Spine J 2007;16:669–78. 17115202 10.1007/s00586-006-0254-1 PMC2213543

[19] Miyazaki M Hymanson HJ Morishita Y Kinematic analysis of the relationship between sagittal alignment and disc degeneration in the cervical spine. Spine 2008;33:E870–876. 18978580 10.1097/BRS.0b013e3181839733

[20] Guo Q Ni B Yang J Relation between alignments of upper and subaxial cervical spine: a radiological study. Arch Orthop Trauma Surg 2011;131:857–62. 21274548 10.1007/s00402-011-1265-x

[21] Berjano P Bassani R Casero G Failures and revisions in surgery for sagittal imbalance: analysis of factors influencing failure. Eur Spine J 2013;22 (suppl 6):S853–8. 24061972 10.1007/s00586-013-3024-x PMC3830040

[22] Charosky S Moreno P Maxy P Instability and instrumentation failures after a PSO: a finite element analysis. Eur Spine J 2014;23:2340–9. 24748413 10.1007/s00586-014-3295-x

[23] Luca A Lovi A Galbusera F Revision surgery after PSO failure with rod breakage:a comparison of different techniques. Eur Spine J 2014;23 (suppl 6):610–5. 25238797 10.1007/s00586-014-3555-9

[24] Berjano P Bassani R Casero G Failures and revisions in surgery for sagittal imbalance: analysis of factors influencing failure. Eur Spine J 2013;22:S853–8. 24061972 10.1007/s00586-013-3024-x PMC3830040

[25] Wang XX Wang LM Wang WD Occipitocervical fusion angle and lower cervical spine degeneration in patients with craniocervical junction malformation. Zhongguo Zuzhi Gongcheng Yanjiu 2014;18:613–8.

[26] Riel RU Lee MC Kirkpatrick JS Measurement of a posterior occipitocervical fusion angle. J Spinal Disord Tech 2010;23:27–9. 20072038 10.1097/BSD.0b013e318198164b

